# Treatment of Severe Poison Ivy: A Randomized, Controlled Trial of Long Versus Short Course Oral Prednisone

**DOI:** 10.14740/jocmr1855w

**Published:** 2014-09-09

**Authors:** Gabrielle Curtis, Amy C. Lewis

**Affiliations:** a Cox Family Medicine Residency, 1423 North Jefferson, Springfield, MO 65802, USA; b Breech School of Business, Drury University 900 North Benton Avenue, Springfield, MO 65802, USA; c Primary Care Health Improvement Project Practice Based Research Network, 1423 North Jefferson, Springfield, MO 65802, USA

**Keywords:** Toxidendron, Poison ivy, Contact dermatitis, Steroid taper

## Abstract

**Background:**

Toxidendron (poison ivy, oak, and sumac) contact dermatitis is a common complaint in the outpatient primary care setting with little evidence-based guidance on best treatment duration.

**Methods:**

This randomized, controlled trial examined the efficacy and side effects of a 5-day regimen of 40 mg oral prednisone daily (short course) compared to the same 5-day regimen followed by a prednisone taper of 30 mg daily for 2 days, 20 mg daily for 2 days, 10 mg daily for 2 days, and 5 mg daily for 4 days over a total of 15 days (long course) in patients with severe poison ivy dermatitis.

**Results:**

In 49 patients with severe poison ivy, non-adherence rates, rash return, medication side effects, and time to improvement and complete healing of the rash were not significantly different between the two groups. Patients receiving the long course regimen were significantly less likely to utilize other medications (22.7% vs. 55.6%, P = 0.02, number needed to treat 3.05).

**Conclusions:**

This study suggests that a longer course prescription may save patients’ time and exposure to excess medication in the treatment of severe poison ivy. Application of this information to clinical practice will save return visits and reduce excess non-prescription medication administration to individual patients.

## Introduction

Contact dermatitis, particularly from Toxidendron foliage (poison ivy, oak, and sumac), is a common complaint in primary care offices. Oral corticosteroids are often used for treatment, but no randomized controlled trials have been found supporting a particular dosing regimen [1-6]. Several recommended regimens exist in the current literature [1, 2, 6-9]. It is commonly thought that too short a treatment course allows for rebound dermatitis after initial improvement [6, 7, 9-11]. Expert opinion and one case report on the commonly prescribed Medrol Dosepak^®^ (total of 84 mg of methylprednisolone tapered over 6 days) note this regimen to be insufficient and likely to cause rebound rash [6, 10]. Expert recommendation for treatment in this area includes use of oral steroids for severe cases [12], variably described as either involving greater than 20% of body surface area, the presence of severe blistering or itching, or involvement of the face, hands, or genital area [4, 9]. Practice patterns within the supporting practice-based research network (PBRN) varied widely from short course doses of steroids to long course doses inclusive of a taper leading us to question the evidence base behind both regimens. Upon finding little literature support and no clear evidence of which method of treatment was more effective, we undertook this study. Our study addressed the question of whether a 5-day regimen of corticosteroid therapy at doses higher than a Medrol Dosepak^®^ is as effective as the same regimen followed by a tapering dose of corticosteroids for initial control and treatment of symptoms (as evidenced by whether the rash improved with study treatment and lengths of time to improvement and resolution of rash, as well as use of other medications for treatment after initiation of the study protocol); compliance and side effects with the study protocol; and prevention of rebound rash from severe poison ivy dermatitis. These were the initial and only study questions identified and reviewed for face validity prior to study initiation by approximately 20 physician members of the Primary Care Health Improvement Project (PCHIP) PBRN and all studied measures are reported below.

## Materials and Methods

We conducted a randomized, controlled trial of a 5-day regimen (short-course arm) of oral prednisone (40 mg daily and 200 mg total per patient) compared to the same regimen followed by a taper (long-course arm) of 30 mg daily for 2 days, 20 mg daily for 2 days, 10 mg daily for 2 days, and 5 mg daily for 4 days (15 days total administration time and 340 mg total per patient) evaluating 49 patients with severe contact dermatitis from poison ivy. Patients from the practices of participating physicians in the PCHIP PBRN were enrolled in the study at the time of initial contact with their primary care provider while seeking treatment for severe poison ivy dermatitis.

Severe poison ivy is diagnosed when patients have clear exposure and consistent rash or rash and known history of reaction PLUS, one of the following: 1) rash > 20% body surface area; 2) rash on hands, feet, face or genitals; 3) involvement of two or more body areas.

A consistent rash is defined as one that is pruritic, burning or irritating on skin directly exposed or in contact with exposed clothing or hand transfer; or a linear rash with vesicles and a history of reaction to poison ivy in the past.

The goal of treatment with both groups was the resolution of symptoms. Inclusion criteria were age 14 or greater, the ability to give informed consent (informed consent could also be obtained from a legal guardian) and a rash consistent with severe poison ivy. Exclusion criteria for this study included the following: age less than 14 years, rash inconsistent with severe poison ivy, recent steroid exposure (within 2 weeks), contraindication to taking oral steroids, and immunosuppression for any reason.

Enrolled study participants were asked to answer the following questions in their follow-up questionnaire, either by mail or telephone. 1) Did the rash get better with the medication? 2) How many days after starting the medication did it take for the rash to start to improve? 3) Did you have to call your doctor or use other medications to get help clearing the rash after enrollment in the study? If so, what medications did you use? 4) How long did it take to go away entirely? 5) Did you complete the course of medication as prescribed? 6) Did you have any side effects that caused you to stop the medication? If so, what were they? 7) Did the rash come back? 8) If the rash returned, was it in the same place?

Randomization occurred at the time of the visit, with each participating office having been provided with packets which contained pre-printed prescriptions. Each patient was provided with a pre-stamped questionnaire to return to our office 1 month after their original office appointment. Enrollees were contacted via telephone if their questionnaires were not received in the expected time frame. We did not evaluate the return rate of appointments for routine recheck due to wide practice variance and this not being necessary for standard of care.

### Statistical analysis

Statistical evaluation was completed using IBM SPSS Statistics 19 on a per-protocol basis via Chi-square analyses, Fisher’s exact probability testing, or two-sample *t*-tests for independent samples as appropriate. Since this was an exploratory study, we did not correct for multiple tests, but all tests that were conducted are reported. This study was approved by the CoxHealth Institutional Review Board and no post-hoc analyses were undertaken.

## Results

**Figure 1:**
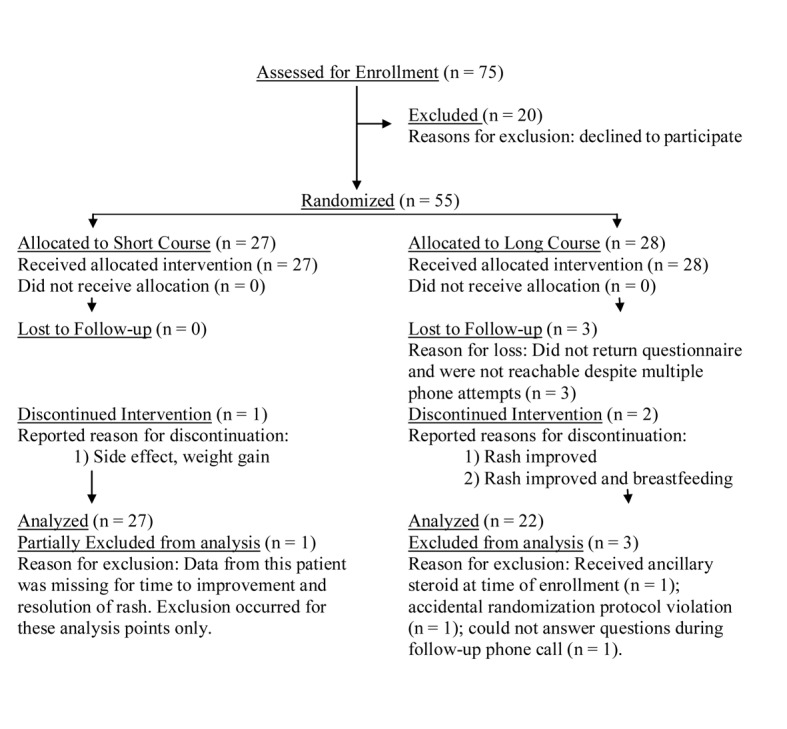
Patients enrollment flowchart.

Information was initially collected from 55 patients meeting criteria for severe poison ivy from April 1, 2009 through December 1, 2009. Forty-nine of these initial patients completed the study. Enrollment flow of patients into the study can be visualized in Figure 1; patients discontinuing intervention were still included in the final analysis. Patient demographics are delineated in Table 1.

**Table 1:** Patients Demographics

	Short course	Long course	Total
Male	7	6	13
Female	20	16	36
Age mean	40.89	34.09	37.88 (SD 17.45, range 15 - 86)
Enrollment			
April	1	3	4
May	3	3	6
June	9	5	14
July	4	3	7
August	7	2	9
September	3	6	9

At the time of questionnaire receipt or phone call, five of 27 in the short-course arm and one of 22 in the long-course arm reported no improvement with the study treatment. However, no significant difference was found between the groups in compliance with the study treatment, overt improvement of rash, time to improvement of the rash, total number of days to complete resolution, or occurrence of side effects, as can be seen in Table 2. Of the three patients complaining of side effects, only one stopped treatment secondary to weight gain. Other reported side effects not leading to discontinuation of medication included anger, hyperactivity, insomnia, and nausea. Similarly, no difference was seen between the groups in reoccurrence of rebound rash. One case of recurring rash was located differently from the original rash, making it unclear if it represented a true rash rebound or a new exposure to poison ivy.

**Table 2:** Clinical Outcomes

	Short course/27	Long course/22	Total/49	P value	Mean difference
Questionnaire	18	15	33		
Telephone	9	7	18		
Noncompliance reported	1	2	3	0.581	
Reported improvement of rash	22	21	43	0.204	
Mean time to improvement	4.42 days (SD 4.13 days)	2.93 days (SD 1.23 days)		0.109	-1.49 days
Mean time to resolution	14.63 days (SD 8.87 days)	11.7 days (SD 7.39 days)		0.23	-2.93 days
Rash return?	4	3	7	0.91	
Return in same location?	4	2	6		
Side effects	3	0	3	0.239	
Use of other medication:	15	5	20	0.02	

Patients receiving the long-course regimen were significantly less likely to utilize other medications (22.7% vs. 55.6%, P = 0.02, number needed to treat 3.05). Additional treatments utilized by both groups as well as statistical significance calculations for this study question can be seen in Table 3. No comparisons other than those listed were originally identified, collected or analyzed in the statistical analysis of these data.

**Table 3:** Statistical Significance of Use of Extra Medications

	Short course/27	Long course/22	Total/49	P value	95% confidence intervals
Use of other medication:	15	5	20	0.02	
Prednisone, Rx	9	2	11		
Depo Medrol, Rx	1	0	1		
Triamcinolone, Rx	1	0	1		
Calamine, OTC	2	4	6		
Antihistamine, OTC	2	2	4		
Hydrocortisone cream, OTC	3	0	3		
Gold bond lotion, OTC	1	0	1		
Other lotion, OTC	2	0	2		
Event rate	22.70%	55.60%			
Absolute risk reduction	32.90%				0.139 - 0.385
Relative risk reduction	59.20%				0.331 - 0.611
Number needed to treat	3.05				2.60 - 7.19
Relative risk	0.41				0.18 - 0.95
Odds ratio	0.24				0.067 - 0.824

## Discussion

Contact dermatitis from Toxidendron (poison ivy, oak, and sumac) is a frequently diagnosed condition in the outpatient primary care setting. Optimal treatment strategy demands provision of cure with maximum reduction in side effects. Expert recommendation has previously been the highest level of evidence found for tapering steroid therapy. Our study suggests that patients who do not utilize a longer treatment course/prescription taper use more medications in addition to the prescription received, which may influence the later development of a rebound rash. An in-depth literature review through OVID utilizing search terms including contact dermatitis, Toxidendron, poison ivy, and steroids/glucocorticoids for treatment revealed this study to be the only randomized, controlled trial to examine the efficacy and side effects of a short-course daily regimen of oral prednisone compared to the same regimen followed by a steroid taper. Our study is limited by small sample size (leading to lower statistical power), and a non-blinded protocol (use of a placebo taper was not feasible within our network resources). The small sample size was the result of a strict adherence to the diagnosis of severe contact dermatitis - all patients in all participating research network practices identified at the time of initial contact with their provider were enrolled over one full poison ivy season. Small sample size can potentially increase the risk of a false positive result. Since this was an exploratory study, we did not correct for multiple tests, but all tests that were conducted were reported.

Despite these limitations, our study suggests that a taper prevents the use of significantly more additional medications, with a relatively low number needed to treat of 3.05. Seventy-five percent of those patients using extra medications came from the short-course arm (15 of 20), and the majority of those patients required extra prescription medication in the form of a longer course of prednisone, intramuscular steroids, or topical steroids. Adding a taper to a prednisone prescription at the time of the initial office visit has the benefits of convenience for the patient and less work output in the form of repeat phone calls, visits, and nursing/physician time. While the non-blinded nature of our study is a limitation and may have prompted patients in the short-course arm to ask for more medication because they knew the other study arm was receiving an extra amount of steroid, we assumed that patients would return only if they had discomfort or symptoms worrisome enough to them to make taking a medication worth the time and trouble to do so.

To enhance power and effect size, larger randomized, controlled studies are needed, specifically to address the magnitude of effect of extra medication utilization in the prevention of rebound rash. The use of extra over the counter and prescription steroids could then be studied individually. In addition, while the use of topical steroids is recommended as A-level evidence for mild contact dermatitis [2], treatment options for cases that are more serious but do not yet meet criteria for severe dermatitis are less well-defined and optimal dosing is unknown.

## References

[1] Usatine RP, Riojas M (2010). Diagnosis and management of contact dermatitis. Am Fam Physician.

[2] Beltrani V, Berstein I, Cohen D, Fonacier L (2006). Contact dermatitis: a practice parameter. Contact dermatitis: a practice parameter. Allergy Asthma Immunol.

[3] Hachem JP, De Paepe K, Vanpee E, Bogaerts M, Kaufman L, Rogiers V, Roseeuw D (2002). Efficacy of topical corticosteroids in nickel-induced contact allergy. Clin Exp Dermatol.

[4] Li LY, Cruz PD, Jr (2004). Allergic contact dermatitis: pathophysiology applied to future therapy. Dermatol Ther.

[5] Saary J, Qureshi R, Palda V, DeKoven J, Pratt M, Skotnicki-Grant S, Holness L (2005). A systematic review of contact dermatitis treatment and prevention. J Am Acad Dermatol.

[6] Wooldridge WE (1990). Acute allergic contact dermatitis. Acute allergic contact dermatitis. Postgrad Med.

[7] Spector SL (1992). Oral steroid therapy for asthma and contact dermatitis. JAMA.

[8] Klaus MV, Wieselthier JS (1993). Contact dermatitis. Am Fam Physician.

[9] Craig K, Meadows SE (2006). What is the best duration of steroid therapy for contact dermatitis (rhus)?. J Fam Pract.

[10] Ives TJ, Tepper RS (1991). Failure of a tapering dose of oral methylprednisolone to treat reactions to poison ivy. JAMA.

[11] Brodell R (1999). How much steroid for poison ivy?. Postgrad Med.

[12] Wolf K (2008). Fitzpatrick's Dermatology in General Medicine.

